# Deubiquitylase OTUD6B stabilizes the mutated pVHL and suppresses cell migration in clear cell renal cell carcinoma

**DOI:** 10.1038/s41419-021-04135-3

**Published:** 2022-02-02

**Authors:** Kai Guo, Yinghua Wei, Ze Wang, Xiaoli Zhang, Xin Zhang, Xinxin Liu, Wenyong Wu, Zhengsheng Wu, Lingqiang Zhang, Chun-Ping Cui

**Affiliations:** 1 Department of General Surgery, The First Affiliated Hospital of Anhui Medical University, Hefei, Anhui People’s Republic of China; 2 Department of Pathology, School of Basic Medical Sciences, Anhui Medical University, Hefei, Anhui China; 3 State Key Laboratory of Proteomics, National Center for Protein Sciences (Beijing), Beijing Institute of Lifeomics, 100850 Beijing, China

**Keywords:** Ubiquitylation, Renal cell carcinoma

## Abstract

Von Hippel-Lindau (VHL) is an important tumor suppressor, and its inactivation is a hallmark of inherited VHL disease and most sporadic clear cell renal cell carcinoma (ccRCC). VHL protein (pVHL) with missense point mutations are unstable and degraded by the proteasome because of the disruption of elongin binding. Deubiquitylase ovarian tumor domain-containing 6B (OTUD6B) had been documented to couple pVHL and elongin B to form stable VHL - elonginB - elonginC complex, which protects pVHL from degradation. However, whether OTUD6B governs the stability of pVHL wild type and the missense mutants in ccRCC remains largely elusive. Here, we reported that low OTUD6B level predicted poorer survival in ccRCC patients with *VHL* missense mutation, but not frameshift deletion and nonsense mutation. OTUD6B is able to interact with wild type pVHL and tumor-derived pVHL missense mutants, except for pVHL I151T, and decrease their ubiquitylation and proteasomal degradation in ccRCC cells. Functionally, we revealed that OTUD6B depletion enhanced cell migration and HIF-2α level in ccRCC cells in a pVHL dependent manner. In addition, OTUD6B depletion reduced the inhibitory effects of ectopic pVHL missense mutants on cell migration and HIF-2α level, except for pVHL I151T. Thus, we speculated that I151 residue might be one of key sites of pVHL binding to OTUD6B. These results suggested that OTUD6B is an important regulator for the stability of pVHL missense mutants, which provides a potential therapeutic strategy for ccRCC with *VHL* mutations.

## Introduction

The von Hippel-Lindau (VHL) tumor suppressor is responsible for substrate recognition in the Cullin-RING ubiqutin ligase complex, which is composed of Cullin 2, elongin B, elongin C, and Rbx1, entitled as Cul2-elonginB/C (CBC) complex [1–4]. Hypoxia inducible factor (HIF) - α, which keeps the balance of oxygen homeostasis with pleiotropic effects, is mainly degraded through the proteasome after VHL-promoted ubiquitin conjugation [5]. Under normoxic condition, proline residues in the oxygen-dependent degradation domains of HIF-α are hydroxylated by proline hydroxylases, then these residues are recognized by pVHL, leading to ubiquitination and subsequent proteasomal degradation of HIF-α [6–8].

Inactivation of *VHL* gene is a hallmark of inherited *VHL* syndrome [6] and sporadic clear cell renal cell carcinoma (ccRCC) [9, 10]. *VHL* disease, resulted from germline mutations in *VHL* gene, is an autosomal dominant disease and predisposes to highly vascularized tumors, and the most frequent manifestations are hemangioblastomas of the central nervous system and retina, ccRCC, and pheochromocytomas [11]. Germline *VHL* missense mutation is the most common kind found in Type 2 form of VHL disease, accounting for 52% of all VHL-related mutations [11, 12]. In addition, up to 80% of ccRCC have the inactivated *VHL* gene [13, 14] which leads to HIF-α accumulation and the activation of HIF target genes which promote tumor angiogenesis, invasion, metabolic reprogramming, and metastasis [15, 16]. Therapies targeting HIF indirectly, such as vascular endothelial growth factor (VEGF) inhibitors, are the first-line treatments for ccRCC, but most patients develop drug resistance [17, 18]. So, it is essential to explore the mechanisms underlying pVHL inactivation for the development of potential therapeutic strategy for ccRCC.

Previous structural studies confirmed that pVHL, elonginB and elonginC formed the ternary complex together [2, 4]. The entire pVHL-elongin B/C complex is exempt from proteasomal degradation, indicating wild-type pVHL can be directly stabilized by binding to elonginB/C [19, 20]. However, pVHL harboring missense point mutations which disrupt the binding with elonginC is unstable and liable to be degraded by the proteasome [9, 10, 13]. Most of the missense mutations concerned with *VHL* syndrome and ccRCC are located in the elonginC - binding domain of pVHL [21, 22]. Many of tumor-derived mutations have been proved to disrupt interaction with elongin [23, 24]. So, loss of elongin binding capability caused by missense mutations promotes tumorigenesis by impairing pVHL stability.

In our recent study, we reported a novel mechanism underlying the regulation of pVHL stability. We found that deubiquitylase ovarian tumor domain-containing 6B (OTUD6B) induced the inactivation of HIF pathway by enhancing the stability of pVHL, and suppressed hepatocellular carcinoma (HCC) metastasis [25, 26]. Further we documented that OTUD6B removed the ubiquitin (Ub) conjugation from pVHL in an OTU independent manner. OTUD6B interacts with wild-type pVHL and elongin B subunits to form more s\CBC^VHL^ ligase complex, protecting pVHL from proteasomal degradation. However, whether OTUD6B governs the stability of pVHL and the missense mutants in ccRCC remains largely elusive.

Here, we reported that low OTUD6B level predicted poorer survival in ccRCC patients with *VHL* missense mutation, but not frameshift deletion and nonsense mutation. OTUD6B is able to interact with wild type pVHL and tumor-derived pVHL missense mutants, except for pVHL I151T, and decrease their ubiquitylation and proteasomal degradation in ccRCC cells. Functionally, we revealed that OTUD6B depletion enhanced cell migration and HIF-2α level in ccRCC cells in a pVHL dependent manner. In addition, OTUD6B depletion reduced the inhibitory effects of ectopic pVHL missense mutants on cell migration and HIF-2α level, except for pVHL I151T. We speculated that I151 residue might be one of key sites of pVHL binding to OTUD6B. Altogether, these results suggested that OTUD6B is an important regulator for pVHL missense mutants, which provides a potential therapeutic strategy for ccRCC with VHL mutations.

## Materials and methods

### Cell culture and cloning procedures

HEK293T, ACHN, CAKI-1, A498, 786O, OSRC-2, 769P were maintained in DMEM, MEM, MCCOY’S 5A, RPMI 1640, which contained 1% penicillin-streptomycin and 10% fetal bovine serum (FBS).

### Lentivirus packaging and infection

In order to construct lentivirus shRNA against human OTUD6B, targeted shRNA sequences were cloned into the pCDH-puro vector. The relative sequences are listed as followed. OTUD6B No. 1: 5′ -GGTATTGACCGAAGAGCTTGA- 3′; No. 2: 5′ -GCTGAGAAGGCATCGCAAAG- 3′. pCDH, pSPAX.2, and pMD.2 G were transfected altogether into HEK293T cells, and cell supernatant was collected every 12 h since the first 24 h after transfection. The cells mentioned above were infected with collected viruses. 48 h later, puromycin was added to the medium of ccRCC cells for selecting positive clones. Those cells with OTUD6B stable knockdown were confirmed through immunoblotting.

### Cell transfections, immunoprecipitation, and immunoblotting

Cells were transfected with various constructs in the presence of high efficiency transfection reagent (Genestar) in accordance with the protocol offered by the manufacturer. With regard to immunoprecipitation assays, HEPES lysis buffer supplemented with protease-inhibitor cocktail (Biotool) was used to lyse cells. Indicated antibodies and protein A/G agarose beads (Santa Cruz) were used to conduct the immunoprecipitation assays at 4 °C. Then wash the immunocomplexes with HEPES by three times. Both lysates and immunoprecipitates were detected with the indicated primary antibodies and related secondary antibodies then the Western Bright ECL chemiluminescent Detection Reagent (Advansta). Supplementary Table 1 includes all the primary antibodies.

### Ubiquitylation assay in cells

HA-Ub and flag-pVHL plasmids were transferred into 293T cells. MG132 was used to treat cells for 8 h before collection. Then the co-immunoprecipitation was conducted with relative tagged antibody, and western blotted with corresponding primary antibody.

### Polymerase chain reaction

Total cellular RNA was extracted using Trizol reagent (Invitrogen) according to the instructions.The cDNA was synthesized by taking 5 micrograms of total RNA and reverse-transcribed using either Toyobo or Revertaid first strand cDNA synthesis kit (Thermo). The reverse transcriptate was 1 µl, each primer was 250 nM, and the volume reaction was 25 µl. Primers for the gene products used are shown in Supplementary Table 2.

### Cell migration assays

Cell migration assay was performed on a 24-well Transwell plate and the upper and lower culture chambers were separated by an 8 μm polyethylene terephthalic acid membrane filter (Corning). Briefly, cells were plated in the upper chamber at 2 × 10^4^ cells per well in serumfree MEM medium. The lower chamber contains MEM medium with 10% FBS. The cells migrated in a wet chamber at 37 °C and 5% CO_2_ for 24 h. At the end of the incubation period, remove the filter and use a cotton swab to separate the nonmigratory cells above the filter. The filters were fixed with 4% formaldehyde for 10 min, and the cells in the lower filter were stained with 0.1% crystal violet for 5 min and photographed.

### Cell proliferation assays

Cell Counting Kit 8 (WST-8/CCK8) (Bimake) was used for cell proliferation assay, WST-8 tetrazolium salt is reduced by cell dehydrogenase to an orange formaldehyde soluble in tissue culture medium. Cells were plated in 96-well plates (100 µl cell suspensions, 3 × 10^4^ cells /ml). After 24 h, 10 μl CCK8 was added to each well, incubated at 37 °C for 4 h, and the absorbance was determined at 450 nm. The values were standardized to wells containing media alone.

### Protein half-life assay

pVHL and OTUD6B plasmids were transferred into 293T cells. After 24 h, the cycloheximide (CHX) (Sigma, 10 µg/ml) was used to treat with the cells for 0, 4, 8 or 12 h before collection.

### Statistical analysis

All the statistics were analyzed by the statistical package for social science. Student’s *t* test, two-way ANOVA test or Wilcoxon Mann–Whitney tests were used. *P* values <0.05 were considered significant.

## Results

### Low OTUD6B level predicted poor survival in ccRCC patients with VHL missense mutation

**Fig. 1:**
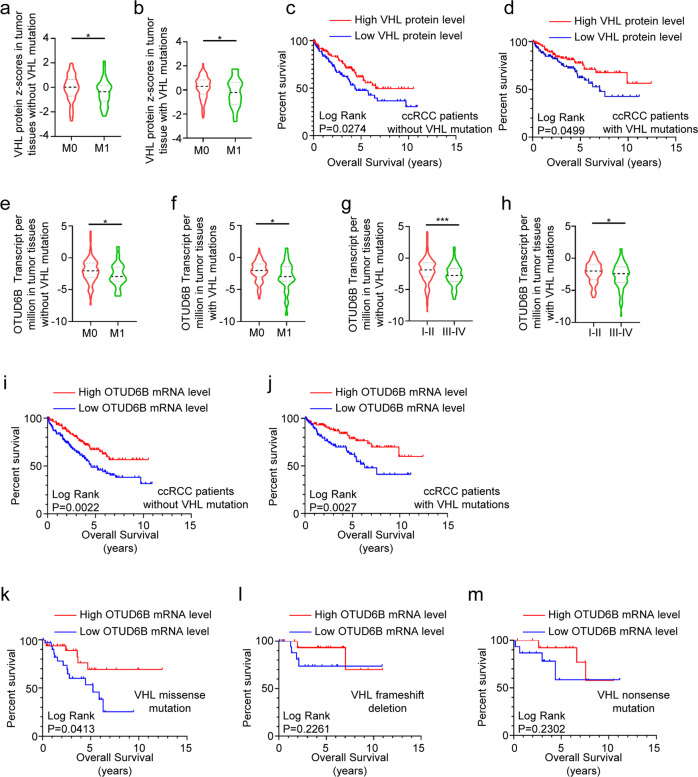
Low OTUD6B level predicts poor survival in ccRCC. **a–j** The ccRCC samples were grouped into two cohorts, VHL wild type and VHL mutation. **a, b** Relative VHL protein level in the tumor tissues of ccRCC patients with stage M0 or M1 were analyzed in the two cohorts respectively. Results are displayed as mean ± s.d. *P < 0.05, Student’s *t* test. **c, d** The Kaplan–Meier curves of pVHL in ccRCC patients with VHL wild type (**c**) or VHL mutations (**d**) for overall survival (OS). **e, f** Relative OTUD6B mRNA levels in the tumor tissues of ccRCC patients with stage M0 or M1 were analyzed in the two cohorts respectively. Results are displayed as mean ± s.d. *P < 0.05, Student’s *t* test. **g, h** Relative OTUD6B mRNA levels in the tumor tissues of ccRCC patients with stage I–II or III–IV were analyzed in the two cohorts respectively. Results are shown as mean ± s.d. *P < 0.05, ***P < 0.001, Student’s *t* test. **i, j** The Kaplan–Meier curves of OTUD6B in ccRCC patients with VHL wild type (**i**) or VHL mutations (**j**) for OS. **k–m** The Kaplan–Meier curves of OTUD6B in ccRCC patients with missense mutation (**k**), frameshift deletion (**l**) and nonsense mutation (**m**) for OS.

Clear cell RCC, the most common type of RCC, is closely associated with *VHL* gene mutations that lead to stabilization of HIF-α in both sporadic and inherited forms [9, 10]. To identify the relationship between pVHL level with the prognosis of ccRCC patients, we analyzed the proteomic results of 471 samples of human ccRCC from The Cancer Genome Atlas (TCGA) dataset. As shown in Fig.1a and b, the higher VHL protein expression was observed in tumor tissue of patients with stage M0. Consistently, high level of pVHL is positively correlated with longer survival of ccRCC patients with or without VHL mutation (*P* < 0.05) (Fig. 1c and d). In addition, we discovered that OTUD6B mRNA level was in a significant decreasing trend with the development of ccRCC pathological stage (including I–II, III–IV stage or M0, M1 stage) in tumor tissue with or without VHL mutation (Fig.1e-h). Next, we analyzed the relationship between OTUD6B mRNA expression and prognosis of ccRCC patients. We divided all the samples into two groups, VHL wild type and VHL mutation, and Kaplan–Meier curves were measured to analyze the correlation of OTUD6B mRNA expression with overall survival (OS). The results suggested that low OTUD6B level indicated a shortened survival time for OS in these two cohorts (Fig. 1i and j). According to the type of VHL mutations, we divided the samples with VHL mutation into three groups, VHL missense mutation, frameshift deletion and nonsense mutation. Interestingly, OTUD6B level is closely related to the OS of ccRCC with VHL missense mutation (*P* = 0.0413) (Fig. 1k), but not the other two cohorts (Fig. 1l and m).

### OTUD6B knockdown enhanced cell migration and reduced VHL protein level in ccRCC cells with wild-type VHL

**Fig. 2:**
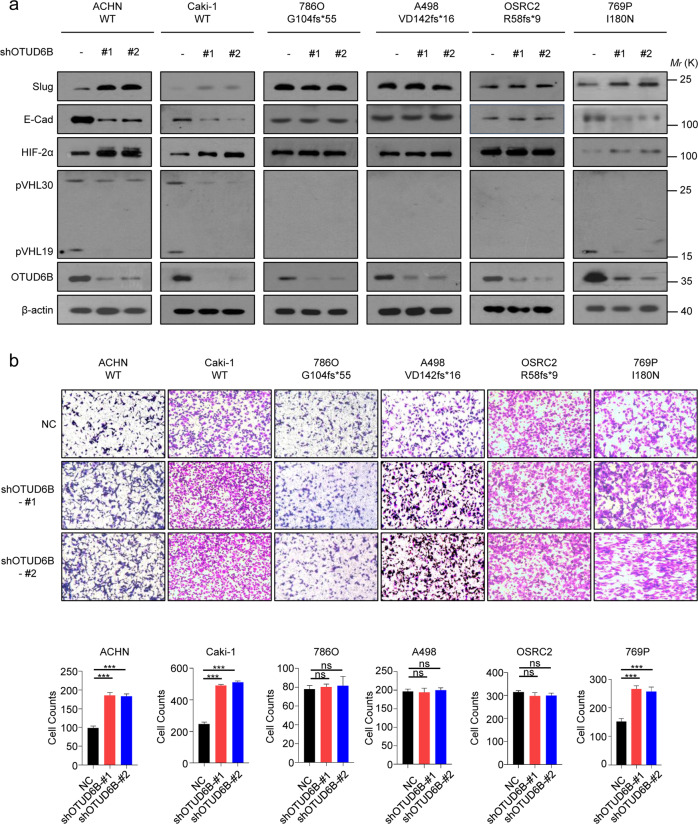
OTUD6B enhances pVHL level and inactivates HIF pathway. **a** The lentivirus with shRNA targeting OTUD6B were each infected into ACHN, Caki-1, 786-O, A498, OSRC2, and 769P cell lines, generating ccRCC cells with OTUD6B stable knockdown. Immunoblot assays were conducted with indicated antibodies in ccRCC cell lines with or without OTUD6B stable knockdown. **b** Transwell assay was conducted to examine cell migration in ACHN, Caki-1, 786-O, A498, OSRC2, and 769P cells with OTUD6B knockdown or negative control (NC). Cell counts were shown in histogram. Results are displayed as mean ± s.d. *n* = 3 independent experiments. ***P < 0.001, Student’s *t* test.

To confirm the functional role of OTUD6B in ccRCC, we knocked down OTUD6B expression in six ccRCC cell lines, including ACHN, Caki-1, 786-O, A498, OSRC-2, and 769P. ACHN and CAKI-1 cells expresses wild type VHL, and 769P cells expresses missense point mutant VHL in which the 180th isoleucine is changed to asparagine (I180N). While in 786-O, A498 and OSRC-2 cells, *VHL* genes have frameshift mutations with G104fs*55, VD142fs*16 and R58f*9 respectively, leading to constitutive HIF-2α expression [27, 28]. Consistently, western blot assay showed that pVHL was undetected in 786-O, A498 and OSRC-2 cells (Supplementary Fig.1a). However, in ACHN, Caki-1 and 769P cells, OTUD6B knockdown markedly decreased pVHL level (Fig. 2a), which was similar to our previous results in HCC cells [25]. We further investigated the interaction between OTUD6B and pVHL in ACHN cells. Endogenous OTUD6B and pVHL were co-immunoprecipitated from lysates of ACHN cells (Supplementary Fig.1 b). Ubiquitylation experiments in ACHN cell lines also showed that OTUD6B inhibited pVHL ubiquitination and improved its stability in ccRCC cells (Supplementary Fig.1c).

**Fig. 3:**
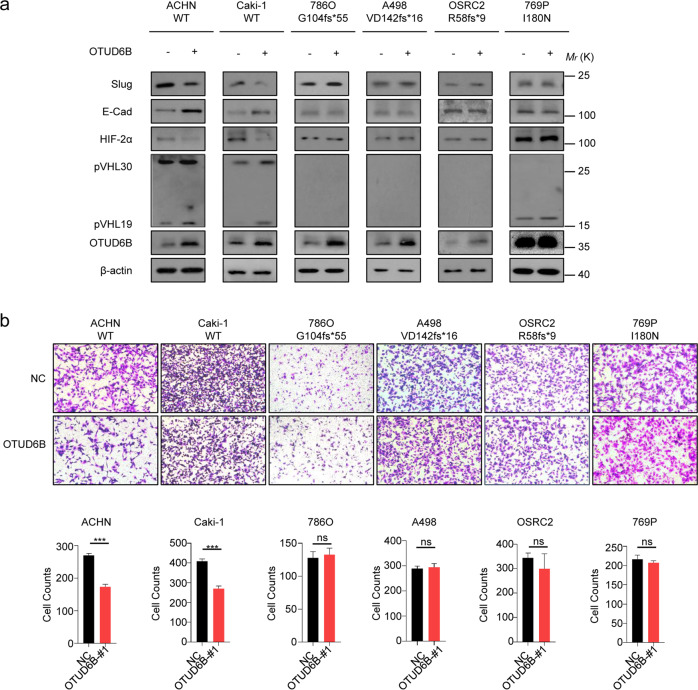
OTUD6B suppresses ccRCC cell migration. **a** The lentivirus with ectopic OTUD6B were each infected into ACHN, Caki-1, 786-O, A498, OSRC2, and 769P cell lines, generating ccRCC cells with OTUD6B stable overexpression. Immunoblot assays were conducted with indicated antibodies in ccRCC cell lines with or without OTUD6B overexpression. **b** Transwell assay was conducted to examine cell migration in ACHN, Caki-1, 786-O, A498, OSRC2, and 769P cells with OTUD6B overexpression or negative control (NC). Cell counts were shown in histogram. Results are displayed as mean ± s.d. *n* = 3 independent experiments. ***P < 0.001, Student’s *t* test.

Subsequently, we went to determine the impact of OTUD6B on HIF signal pathway. We observed that OTUD6B depletion elevated HIF-2α protein level in ACHN, Caki-1 and 769P cells, but not 786-O, A498 and OSRC-2 cells (Fig. 2a). In addition, we used transwell and CCK8 assays to assess the cell migration and proliferation capacities. As shown in Fig. 2b, the results indicated that OTUD6B depletion significantly promoted cell migration capacity in ACHN, Caki-1 and 769P cells, but not cell lines with *VHL* frameshift mutations. Consistently, in ACHN, Caki-1, and 769P cells with OTUD6B stable knockdown, the protein level of E-Cadherin, the epithelial marker [29], was dramatically reduced, while the level of Slug, one of the mesenchymal markers [30], was increased markedly (Fig. 2a). However, there were no effects observed on cell proliferation (Supplementary Fig. 2c) in all the six ccRCC cell lines, like our previous results in HCC cells [25]. OTUD6B has been reported to regulate cell cycle in non small cell lung cancer cells [31, 32]. We detected the level of Cyclin D1 and Cyclin E in these six ccRCC cell lines, and found that in ccRCC cells, cell cycle was not influenced (Supplementary Fig. 2a). At the same time, we overexpressed OTUD6B in the six ccRCC cell lines and performed the above experiments. Consistently, we found that in ACHN and Caki-1 cell lines ectopic OTUD6B increased pVHL expression (Fig. 3a) and cell migration (Fig. 3a, b), decreasing HIF-2α level (Fig. 3a). However, in 786O, A498, and OSRC-2 cells (Fig. 3a and 3b), the pVHL and HIF -2α level were not affected by ectopic OTUD6B, indicating the effects of OTUD6B were pVHL-dependent in ccRCC cells.

### OTUD6B increased the protein stability of tumor-derived missense mutants of pVHL

**Fig. 4:**
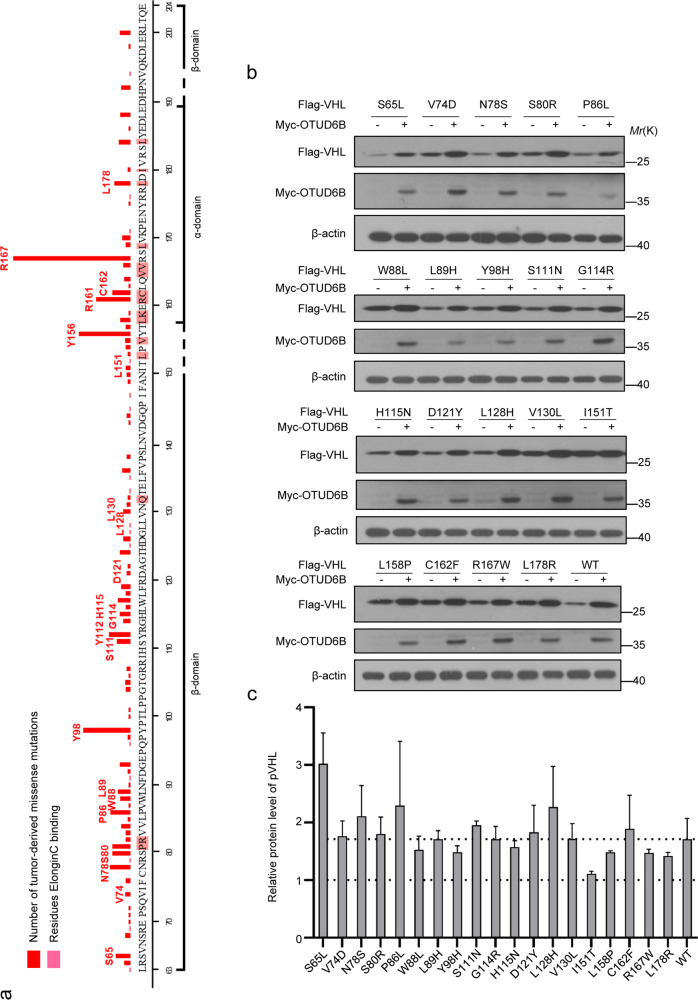
OTUD6B increases the levels of pVHL mutants. **a** Missense mutations were located in α and β domains of pVHL. The histogram represents total of 897 missense mutations records in UMD-VHL mutations database. The 19 most frequently mutated amino acids are labeled. **b** HEK293 cells transfected with indicated pVHL mutants were immunoblotted with anti-flag or anti-myc antibodies. **c** Quantification of relative levels of pVHL and the mutants are shown. The results are expressed as mean ± sd. Each error bar shows the standard deviations of three independent experimental values. ***,*P* < 0.001 compared with the level of wild type pVHL, Student’s *t* test.

**Fig. 5:**
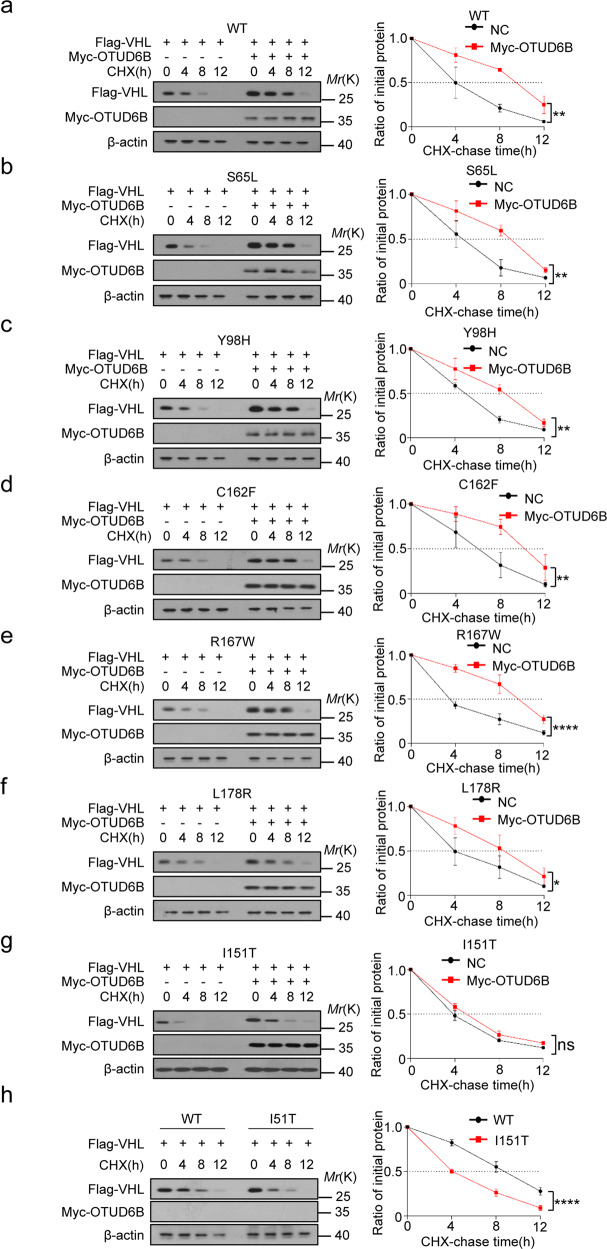
OTUD6B enhances stability of VHL mutants. **a** HEK293 Cells was treated with 10 μg/ml cycloheximide (CHX) for 0, 4, 8, 12 h after transfected with wild type pVHL and OTUD6B for western blot. **b–h** HEK293T treated with 10 μg/ml CHX after transfected with indicated pVHL mutants and OTUD6B was gathered at the indicative hours for immunoblotting. Quantification of pVHL levels relative to β-actin is shown. *P < 0.05, **P < 0.01, ****P < 0.0001, two-way ANOVA test.

Considering the importance of VHL missense mutations in ccRCC, we wonder if OTUD6B generally influences the stability of pVHL missense point mutants associated with inherited or sporadic ccRCC. According to records from the UMD-VHL mutations database (http://www.umd.be/VHL/) [33], total of 239 VHL missense variants were reported. In Fig. 4a, tumor-derived VHL missense mutations map to the α and β domain. More than ten most frequently mutated residues were labeled. Then, we generated nineteen constructs of tumor-derived VHL mutants, including VHL S65L, V74D, N78S, S80R, P86L, W88L, L89H, Y98H, S111N, G114R, H115N, D121Y, L128H, V130L, I151T, L158P, C162F, R167W, and L178R. Then the stability of these pVHL mutants were detected in cells. The results of western blot showed that compared with negative control, the protein levels of wild type pVHL and the majority of these missense mutants were dramatically increased in ectopic OTUD6B overexpressed cells, except for pVHL I151T mutant (Fig. 4b and c). Further, we treated cells with 10 µg/ml CHX to inhibit protein synthesis and examined the half-life of pVHL mutants in cells. As shown in Fig. 5 a–h, ectopic overexpression of OTUD6B markedly prolonged the half-life of pVHL wild type, as well as S65L, Y98H, C162F, R167W, L178R mutants, but not I151T mutants.

### OTUD6B interacted with pVHL tumor-derived missense mutants and reduced their ubiquitylation in cells

**Fig. 6:**
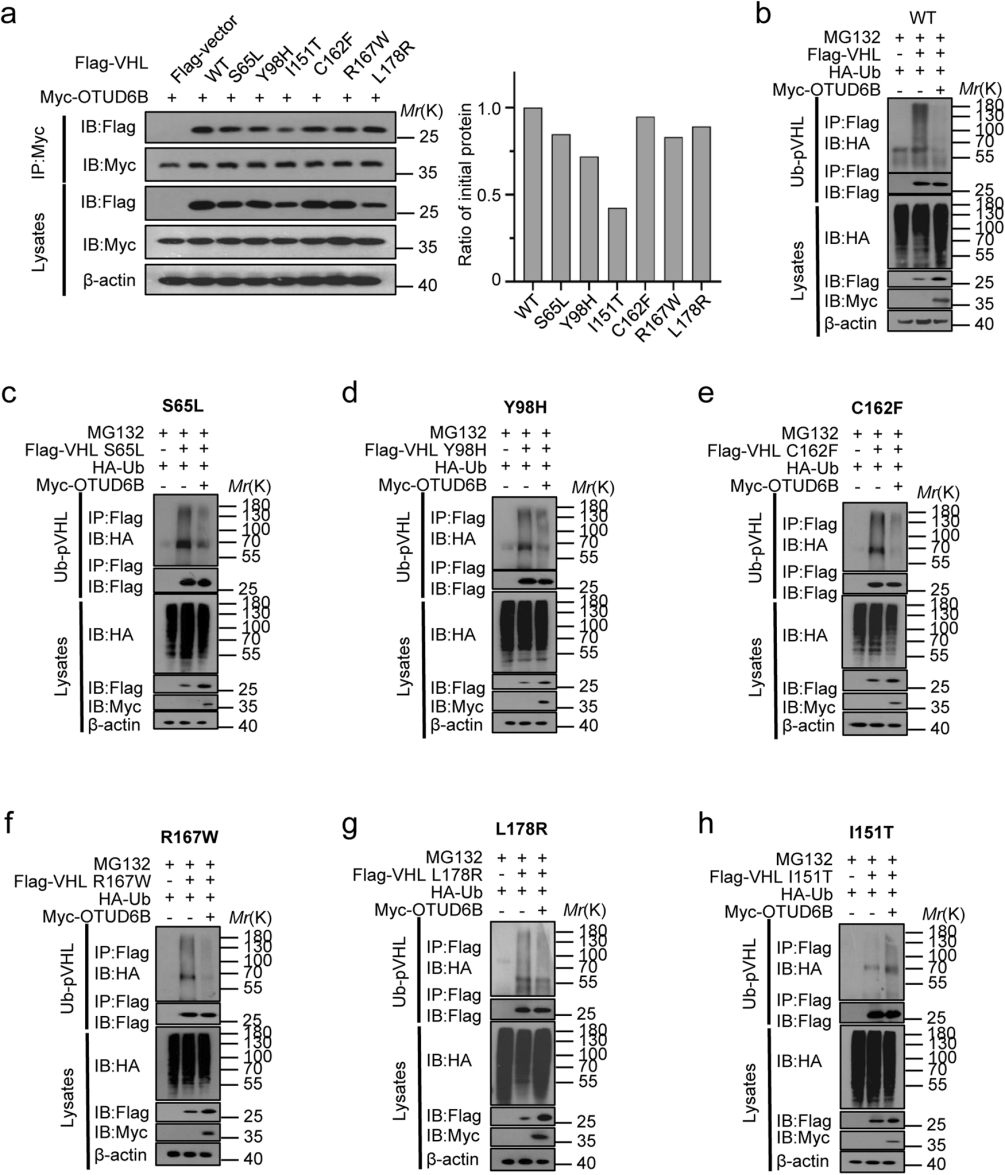
OTUD6B interacts with pVHL mutants and regulates their ubiquitylation. **a** HEK293T was transfected with indicated constructs, the lysate was collected, and then immunoprecipitated with anti-myc antibody and western blotting with either anti-flag or anti-myc antibodies. **b–h** HEK293T was transfected with indicated constructs and treated with MG132 for 8 h. Cell lysates were collected and subject to immunoprecipitation with anti-flag antibody and immunoblotted with indicated antibodies.

We previously reported that OTUD6B reduced ubiquitylation of pVHL in an enzyme independent manner. CBC^VHL^ ligase complex consists of OTUD6B coupling pVHL and elongin B/C, which keeps pVHL stable from being degraded by the proteasome [25]. Here, we tested the interaction between OTUD6B with pVHL mutants. The results showed that ectopic OTUD6B interacted with indicated pVHL mutants in HEK293 cells, but compared with pVHL wild type and the majority of mutants, the binding between pVHL I151T mutant with OTUD6B was markedly weakened (Fig. 6a). In addition, we observed that ectopic OTUD6B suppresses ubiquitylation of majority of pVHL mutants (Fig. 6 b–g), except for pVHL I151T (Fig. 6h).

### Cell migration inhibition induced by ectopic pVHL mutants was blunted by OTUD6B knockdown in 786O cells

**Fig. 7:**
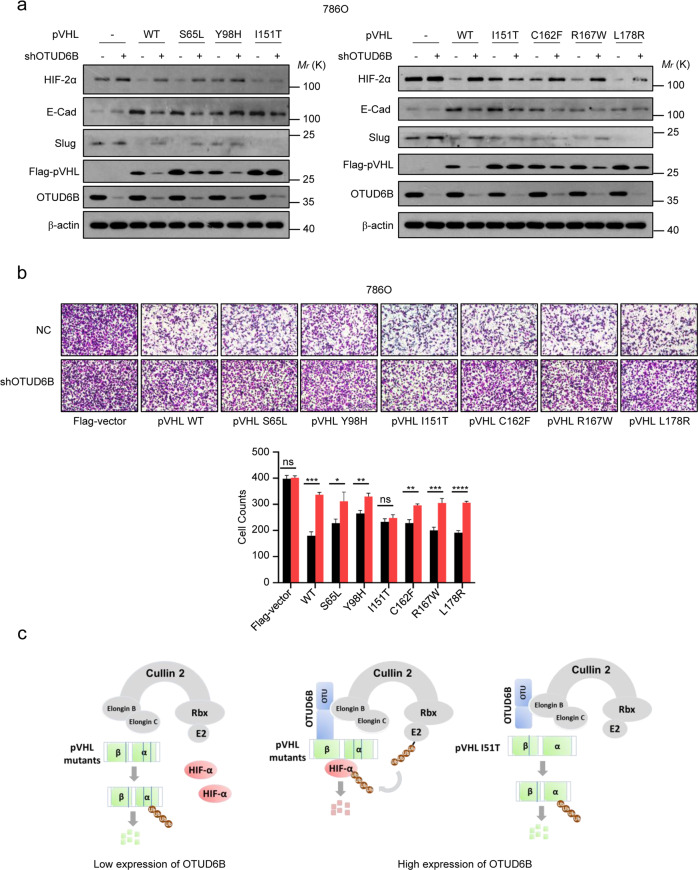
OTUD6B knockdown influences ectopic pVHL and the mutants induced cell migration inhibition. **a** pVHL or the mutant was transfected to 786O cells with OTUD6B stable knockdown. The cell lysates were immunoblotted with indicated antibodies. **b** Transwell assays were conducted in 786O cells. Cell counts were shown in histogram. Results are expressed as mean ± sd. Each error bar shows the standard deviations of three independent experimental values. *P < 0.05, **P < 0.01, ***P < 0.001, ****P < 0.0001, Student’s *t* test. **c** The predicted work model how OTUD6B regulated the stability of pVHL missense mutants.

Given OTUD6B promoted stabilization of pVHL mutants, we ask if OTUD6B recovers the functional role of pVHL mutants in ccRCC cells. We overexpressed pVHL or the indicated mutants in 786O cells with OTUD6B knockdown or NC. Then, western blotting was performed to determine the levels of HIF-2α, E-cadherin and Slug in cells, and transwell assay was conducted to test the capacity of cell migration. Compared with the negative control, ectopic pVHL dramatically decreased HIF-2α level and cell migration in 786O cells. As expected, E-Cadherin and slug, the markers of epithelial-mesenchymal transition (EMT), were upregulated and downregulated respectively. Interestingly, ectopic pVHL mutants including S65L, Y98H, I151T, C162F, R167W, and L178R reduced HIF-2α level and cell migration to varying degrees compared with the negative control, but they were much less active than the wild type pVHL (Fig.7 a and b), suggesting that these missense mutations disrupted the function of pVHL partly. At the same time, we observed that OTUD6B depletion reduced the inhibitory effects of pVHL wild-type and the mutants on cell migration and HIF-2α level, except for pVHL 151T mutant (Fig.7a and b), suggesting that I151 residue might be one of key sites of pVHL binding to OTUD6B.

Taken together, we discovered that OTUD6B increased the stability and activity of both pVHL wild type and missense mutants in ccRCC, which provided a potential therapeutic strategy for ccRCC with VHL mutations.

## Discussion

Renal cell carcinoma (RCC), the incidence of which accounts for 3% of all adult malignancies, is the most prevailing form of kidney cancer [34, 35]. As shown, there has been some improvement in the 5‐year relative survival rates at diagnosis, however, the overall prognosis is still discouraging [35]. ccRCC, the most common subtype of RCC, explains 75% of cases, and is strongly connected with alterations in the *VHL* gene [36]. In addition, although VHL disease associated ccRCC tend to be low grade and minimally invasive [11], up to 70% of VHL patients develop into ccRCC by the age of 60 and ultimately a majority of patients die of ccRCC [37]. In the tumor cells, the inactivation of pVHL leads to accumulation of the HIF and finally to overexpression of VEGF and platelet‐derived growth factor, which promote tumor angiogenesis, invasion, metabolic reprogramming, and metastasis [17]. Understanding the regulatory mechanism of the pVHL and its mutants potentially contributes to the development of targeted therapy. Here we revealed that OTUD6B played as a regulator of the stability of pVHL missense mutants, which provided a potential therapeutic strategy for ccRCC.

Structural and biochemical studies discovered that pVHL forms a ternary complex with the elonginC and elonginB proteins [2–4]. The pVHL-elonginC-elonginB complex is usually disrupted in most of cells with tumor-derived mutations [2, 21, 22, 38]. VHL protein contains two domains: a roughly 100-residue NH_2_-terminal domain rich in β sheet (β domain) and a smaller α-helical domain (α domain), held together by two linkers and a polar interface [2, 4]. The great majority of the α domain surface, and a fraction of the β domain, interact with elongin C, whereas pVHL and elongin B do not interact directly [2, 4]. Pavletich, et al. reported that ~50% of the tumorigenic mutations mapped to the residues that contact elongin C, indicating the pivotal role of the elonginC binding in tumor suppression function of pVHL [39]. Further, Burk et al. found that pVHL with mutations which disrupt elongin binding are liable to be degraded by the proteasome [19, 20]. In contrast, pVHL wild type are stable by interacting with elongin C [19, 20]. In the present study, we consistently observed that compared with wild-type pVHL, the half-life of tumor-derived pVHL missense mutants were significantly shorten.

OTUD6B encodes a member of OTU-containing subfamily of DUBs, several of which were documented to be Ub chain linkage specific [40]. However, the functional role and mechanism of action of OTUD6B remain largely unclear. Recently researchers disclosed that the OTUD6B biallelic pathogenic variant was associated with epileptic seizures and deformities in 12 individuals from six independent families with intellectual disability syndrome [41, 42]. Homozygous OTUD6B knockout mice, which were smaller in size and congenitally defective in heart, were dead at the birth day [39]. We previously reported that OTUD6B promoted the stabilization of wild type pVHL in cells and suppressed HCC metastasis [25, 26]. Furthermore, we observed that OTUD6B was able to stabilize pVHL in a way independent of its DUB enzyme activity. OTUD6B binds to the β-domain of pVHL and elongin B, respectively and enhance the interaction between pVHL and elonginB/C binary complex, protecting pVHL from proteasomal degradation.

In our present study, we further explored the impacts of OTUD6B on the stability of tumor-driven pVHL mutants. Firstly, we disclosed that OTUD6B knockdown increased HIF-2α level and suppressed cell migration in ACHN cells. Using the transcriptome data of human ccRCC samples from TCGA database, we analyzed the relationship between OTUD6B expression and prognosis in ccRCC patients. These results suggested that low level OTUD6B indicated a shorter survival time in these cohorts. Further, according to the type of VHL mutations, we divided the samples with VHL mutation into three groups, VHL missense mutation, frameshift deletion, and nonsense mutation. Interestingly, OTUD6B level is closely related to the OS of ccRCC with VHL missense mutation, but not the other two cohorts. Here we revealed that OTUD6B functioned as a tumor suppressor in ccRCC, which expands our understanding on the functional roles of the OTU DUBs family.

Then, we conducted a series of biochemical experiments to examine the regulatory effects of OTUD6B on pVHL mutants. Ectopic OTUD6B inhibited ubiquitylation and prolonged the half-life of majority of pVHL mutants, while OTUD6B knockdown blunted the effects of pVHL mutants in ccRCC cells, including cell migration inhibition and HIF-2α stabilization, indicating OTUD6B stabilized tumor-derived pVHL missense mutants and promoted their activity in ccRCC cells. Interestingly, we observed that stability of pVHL I151T mutant was not increased by ectopic OTUD6B, and OTUD6B depletion was not able to influence the effects of this mutant in 786O cells. Considering that I151T mutation maps to the β domain, the OTUD6B-binding region of pVHL [25], we proposed that I151 might be one of key sites of pVHL binding to OTUD6B.

Overall, we discovered that OTUD6B was an important regulator for tumor-derived missense mutated pVHL. As shown in Fig.7c, in ccRCC tumor cells with pVHL missense mutations, at absence of OTUD6B, the binding of elonginC and pVHL is disrupted, and pVHL is ubiquitylated and degraded. While at the presence of OTUD6B, OTUD6B couples mutated pVHL binding to elonginC, and protects pVHL mutants from degradation. These findings might provide a potential therapeutic strategy for ccRCC with VHL missense mutations.

## Supplementary information

Supplemental information

Supplementary Table 1

Supplementary Table 2

## Data Availability

All data generated or analyzed during this study are included in this published article and its supplementary information files.
