# Correction to: Deubiquitylase OTUD6B stabilizes the mutated pVHL and suppresses cell migration in clear cell renal cell carcinoma

**DOI:** 10.1038/s41419-022-04970-y

**Published:** 2022-06-01

**Authors:** Kai Guo, Yinghua Wei, Ze Wang, Xiaoli Zhang, Xin Zhang, Xinxin Liu, Wenyong Wu, Zhengsheng Wu, Lingqiang Zhang, Chun-Ping Cui

**Affiliations:** 1 Department of General Surgery, The First Affiliated Hospital of Anhui Medical University, Hefei, Anhui People’s Republic of China; 2 Department of Pathology, School of Basic Medical Sciences, Anhui Medical University, Hefei, Anhui China; 3 State Key Laboratory of Proteomics, National Center for Protein Sciences (Beijing), Beijing Institute of Lifeomics, 100850 Beijing, China

**Keywords:** Ubiquitylation, Renal cell carcinoma

Correction to: *Cell Death and Disease* 10.1038/s41419-021-04135-3, published online 02 February 2022

The original version of this article unfortunately contained mistakes in the affiliations. Affiliation 2 should read “Department of Pathology, School of Basic Medical Sciences, Anhui Medical University, Hefei, Anhui, China”. In addition, author Chun-Ping Cui is also affiliated with “Department of Pathology, School of Basic Medical Sciences, Anhui Medical University, Hefei, Anhui, China”. The original article has been corrected.

