# Supplementary material for: Deubiquitylase OTUD6B stabilizes the mutated pVHL and suppresses cell migration in clear cell renal cell carcinoma
Source: Cell Death Dis. 2022 Feb 2;13(2):97. doi: 10.1038/s41419-021-04135-3 (PMC8810859; doi:10.1038/s41419-021-04135-3)
Supplement: Supplementary file 1 — Supplemental information [file 41419_2021_4135_MOESM1_ESM.docx]

**Supporting Information**

**Deubiquitylase OTUD6B stabilizes the mutated pVHL and suppresses cell migration in Clear Cell Renal Cell Carcinoma**

Kai Guo^1, 2, 3, #^, Yinghua Wei^2, 3, #^, Ze Wang^3^, Xiaoli Zhang^3^, Xin Zhang^3^, Xinxin Liu^3^, Wenyong Wu^1, *^, Zhengsheng Wu^2, *^, Lingqiang Zhang^3, *^, Chun-Ping Cui^3, *^

**
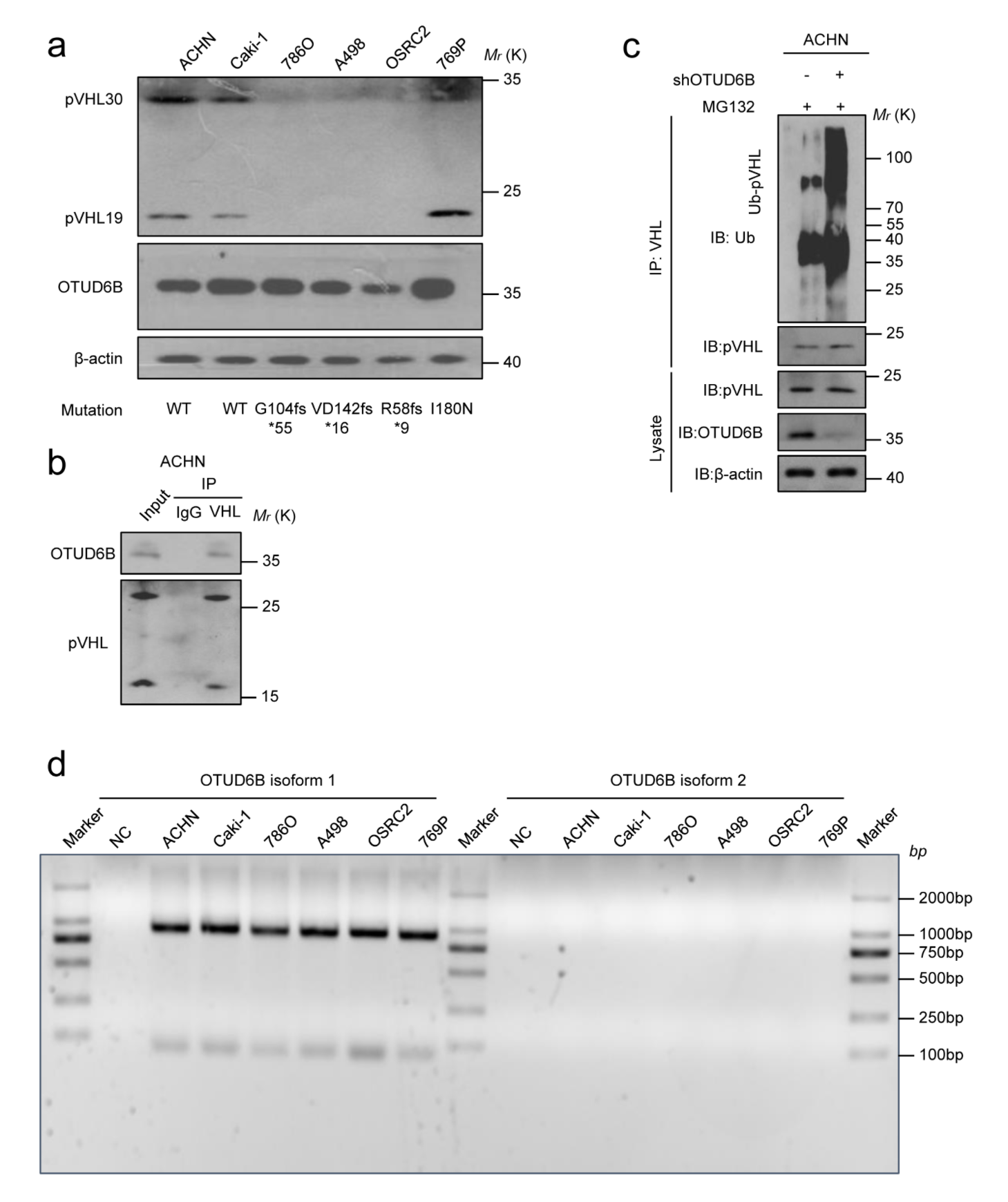
**

**Supplementary Figure 1.** **The expression of OTUD6B in ccRCC cell lines**

**a.** Immunoblot assays were conducted with indicated antibodies in ccRCC cell lines. **b.** ACHN cell lysates were collected then subject to immunoprecipitation (IP) with anti-VHL or control IgG antibodies. **c.** ACHN cells with or without OTUD6B stable knockdown were treated with MG132 for 8 h before collection. Cell lysates were subject to IP assays with anti-VHL or control IgG antibodies subsequently immunoblotted with indicative antibodies. **d.** PCR was performed in ccRCC cells with OTUD6B primers of isoform 1 and isoform 2. The results were shown by agarose gel electrophoresis.

**
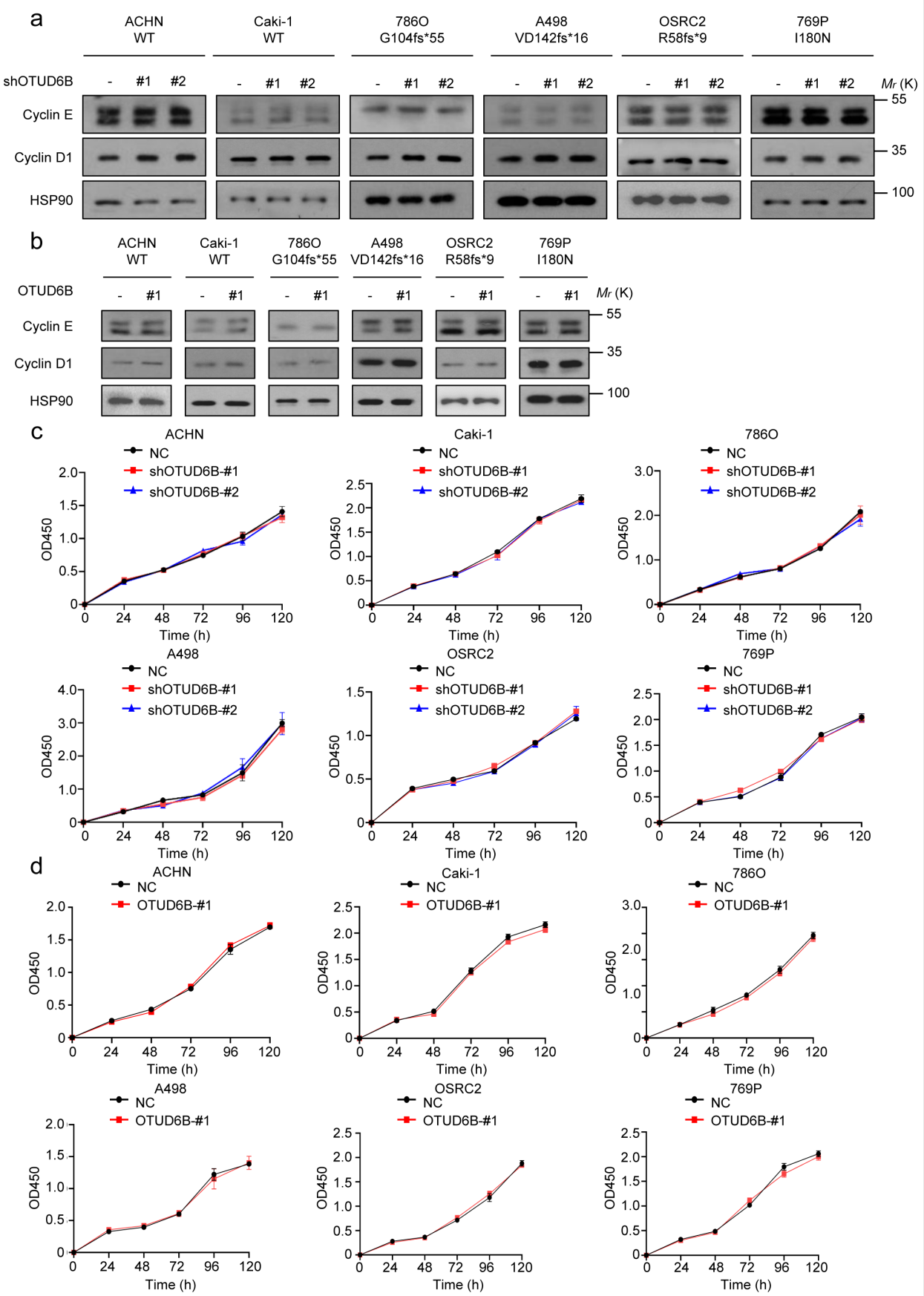
**

**Supplementary Figure 2. OTUD6B has no effects on proliferation of ccRCC cells.**

**a.** Cyclin D and E were examined in ccRCC cells with OTUD6B stable knockdown. **b.** In ccRCC cells with OTUD6B stable overexpression, Cyclin D and E were also detected. **c.** CCK-8 assays were used to examine cell proliferation in indicated cell lines with OTUD6B knockdown or NTC. **d.** CCK-8 assays were used to determine cell proliferation in ccRCC cells with OTUD6B stable overexpression.
